# Multidrug-resistant uropathogens in pediatric urinary tract infections: a multicenter retrospective trend analysis (2020–2024)

**DOI:** 10.1007/s00210-026-05134-x

**Published:** 2026-02-25

**Authors:** Esra Erdoğan, Azize Yetişgen, Serpil Doğan, Muhammed Selçuk Sinanoğlu, Fedli Emre Kılıç, Osman Kurt

**Affiliations:** 1 Department of Pharmaceutical Basic Sciences, Faculty of Pharmacy, Harran University, Şanlıurfa, Türkiye; 2 Infectious Diseases Clinic, Malatya Training Research Hospital, Malatya, Türkiye; 3 Microbiology Clinic, Kahramanmaraş Necip Fazıl City Hospital, Kahramanmaraş, Türkiye; 4 Department of Child Health and Diseases, Faculty of Medicine, Malatya Turgut Özal University, Malatya, Türkiye; 5 Department of Child Health and Diseases, Faculty of Medicine, Adıyaman University, Adıyaman, Türkiye; 6 Department of Public Health, Faculty of Medicine, Inönü University, Malatya, Türkiye

**Keywords:** Pediatric urinary tract infections, Antibacterial resistance, COVID-19 pandemic, Kahramanmaraş earthquake, Gram-negative bacteria, Multidrug-resistant pathogens

## Abstract

Large-scale public health disruptions, including pandemics and natural disasters, may influence healthcare delivery, pathogen distribution, and antimicrobial resistance (AMR). This retrospective multicenter study evaluated uropathogen profiles and temporal trends in antibacterial resistance among hospitalized pediatric patients with urinary tract infections (UTIs) across periods corresponding to the COVID-19 pandemic, the February 6, 2023 Kahramanmaraş earthquakes, and the post-earthquake phase in heavily affected regions of Türkiye. Hospitalized pediatric patients (0–18 years) with culture-confirmed UTIs admitted between January 2020 and December 2024 to three tertiary care hospitals were included. Pathogen identification and antibacterial susceptibility testing were performed using standard microbiological methods in accordance with EUCAST criteria. Uropathogen distribution and resistance patterns were compared across predefined study periods. A total of 1131 pediatric patients were analyzed, of whom 54.29% were female. Gram-negative bacteria predominated (89.57%), with *Escherichia coli* (59.86%) and *Klebsiella *spp. (18.92%) being the most frequently isolated pathogens. Across the study periods, *E. coli* demonstrated significant increases in resistance to ampicillin (63.26% to 81.90%), ceftriaxone (41.99% to 53.76%), ceftazidime (39.46% to 63.10%), and trimethoprim–sulfamethoxazole (32.27% to 40.34%) (all *p* < 0.01). Among *Klebsiella *spp., a significant temporal increase was observed only for imipenem resistance, which rose from 18.64% during the COVID-19 period to 37.50% during the earthquake period before declining to 13.21% in the post-earthquake period (*p* = 0.021). Multidrug-resistant (MDR) phenotypes were predominantly detected among Gram-negative organisms, with the highest proportions observed in Serratia spp.,* Citrobacter* spp., *Enterobacter *spp., and *Pseudomonas *spp., while MDR prevalence in *E. coli* was comparatively lower. Temporal variations in antibacterial resistance were observed among pediatric UTI pathogens during periods of major public health disruption. These findings highlight the importance of sustained regional surveillance and context-aware empiric treatment strategies in settings exposed to systemic healthcare stressors.

## Introduction

Türkiye is located in one of the most seismically active regions in the world and has experienced numerous devastating earthquakes throughout its history. On February 6, 2023, two consecutive earthquakes with magnitudes of 7.7 and 7.6 struck the Pazarcık and Elbistan districts of Kahramanmaraş province along the East Anatolian Fault Line, affecting 11 provinces and resulting in more than 50,000 deaths and over 100,000 injuries. Recorded as the most destructive seismic event in the region since the early 1900 s, the disaster was followed by more than 1,000 aftershocks, further exacerbating infrastructure collapse and population displacement. Approximately 14 million people were directly affected, including an estimated 4.6 million children, who represent one of the most vulnerable groups during and after disasters due to inadequate shelter, disrupted healthcare access, adverse social conditions, and separation from family members (Canpolat et al. 2023; Akbaba et al. 2024; Şenol Balaban et al. 2024).

Following the Eastern Anatolia earthquakes, an increased risk of infectious diseases—particularly those involving multidrug-resistant (MDR) organisms—was observed. Several factors, including damage to healthcare infrastructure, overcrowded living conditions, poor sanitation, and limited access to clean water, may represent potential mechanisms contributing to this increased vulnerability; however, these factors were not directly measured in the present study. These challenges may have been further compounded by the prolonged Syrian conflict and the residual effects of the COVID-19 pandemic, both of which placed sustained pressure on regional healthcare systems. Within this broader context, higher rates of antibacterial-resistant infections were observed among hospitalized patients, warranting concern regarding healthcare-associated transmission and the need for strengthened antibacterial stewardship, rather than implying demonstrated causality (Ahmed et al. 2023; Mavrouli et al. 2023; Okuyama et al. 2024).

Urinary tract infections (UTIs) represent one of the most common bacterial infections in the pediatric population, ranking second only to respiratory infections. It is estimated that approximately 8% of girls and 2% of boys experience at least one UTI by the age of 10 years. Clinical presentation varies with age: older children typically present with dysuria, fever, and abdominal pain, whereas infants may exhibit nonspecific symptoms such as irritability, poor feeding, and failure to thrive. Beyond their clinical impact, UTIs impose a significant economic and healthcare burden due to recurrent infections, hospitalizations, and long-term complications such as renal scarring. *Escherichia coli* (*E. coli*) remains the predominant uropathogen; however, other Gram-negative bacteria, including *Klebsiella spp*. and *Pseudomonas aeruginosa*, as well as Gram-positive organisms such as *Enterococcus spp.* and *Staphylococcus aureus* (*S. aureus*), are increasingly implicated, particularly in hospitalized children and complicated infections (Maringhini et al. 2024; Kılıç and Küçükkelepçe, 2025).

Effective management of pediatric UTIs relies on timely initiation of appropriate antibacterial therapy, taking into account pathogen susceptibility, infection severity, patient age, and prior antibacterial exposure. Current guidelines from the European Association of Urology (EAU) recommend fosfomycin, nitrofurantoin, and pivmecillinam as first-line agents for uncomplicated cystitis, while trimethoprim–sulfamethoxazole or cephalosporins may be considered in settings with low resistance rates. For pyelonephritis, fluoroquinolones or cephalosporins with adequate renal tissue penetration are preferred. To minimize treatment failure and curb the development of resistance, empirical therapy should be promptly revised based on culture results; local antibacterial resistance patterns should guide antibacterial selection (Bonkat et al. 2017; Silva et al. 2022).

Despite advances in diagnostic and therapeutic strategies, UTIs remain a major driver of antibacterial use and a significant contributor to the global burden of antimicrobial resistance (AMR), particularly among bacterial pathogens. UTIs are associated with substantial morbidity, mortality, and healthcare costs, amounting to billions of dollars annually, while the widespread and often repeated use of antibacterial agents for these infections has accelerated the emergence of resistant uropathogens. Although antibacterial therapy remains the cornerstone of UTI management, increasing resistance among urinary isolates has progressively undermined the effectiveness of this approach (Timm et al. 2025). Rising AMR rates, particularly among Gram-negative bacteria, threaten the success of empirical treatment and are further complicated by the growing prevalence of MDR organisms. Treatment options for MDR infections are often limited, especially in pediatric populations, where the number of antibacterials approved for use is restricted. Consequently, the selection of empirical therapy for pediatric UTIs has become increasingly complex, underscoring the need for continuous surveillance of local resistance patterns and the development of alternative, targeted therapeutic strategies (El Zein et al. 2025).

The World Health Organization (WHO) declared COVID-19 a global pandemic in March 2020, leading to widespread public health interventions, including social distancing, mask use, enhanced hygiene practices, and restrictions on movement (Flaxman et al. 2020; Erdogan and Delen 2022). Several studies have reported a decline in pediatric UTI incidence during the pandemic period, likely attributable to improved hygiene, reduced interpersonal contact, and decreased hospital admissions (Ilmavirta et al. 2024; Khavandegar et al. 2025). However, changes in healthcare utilization patterns and antibacterial prescribing practices during this period may have also influenced AMR trends.

Both natural disasters and pandemics have the potential to profoundly alter the epidemiology, clinical management, and AMR patterns of UTIs. The 2023 Kahramanmaraş earthquakes provide a unique opportunity to examine how large-scale infrastructure disruption, compromised hygiene conditions, and healthcare system overload influence healthcare-associated and community-acquired uropathogen distribution and resistance dynamics. Concurrently, the COVID-19 pandemic represents an unprecedented global intervention that reshaped infection transmission patterns and antibacterial use behaviors.

Therefore, this multicenter retrospective study aimed to evaluate changes in uropathogen distribution and AMR patterns among hospitalized pediatric patients aged 0–18 years in the provinces of Adıyaman, Kahramanmaraş, and Malatya between January 2020 and December 2024, encompassing both the COVID-19 pandemic and post-earthquake periods. The findings of this study are intended to inform empirical treatment strategies and infection control policies for pediatric UTIs under extraordinary conditions such as pandemics and natural disasters.

## Methodology

### Study participants

This multicenter retrospective observational study was conducted using medical records from three tertiary care hospitals located in regions severely affected by the 2023 Kahramanmaraş earthquakes: Adıyaman Training and Research Hospital, Kahramanmaraş Necip Fazıl City Hospital, and Malatya Training Research Hospital. The study period covered five years, from January 2020 to December 2024, encompassing both the COVID-19 pandemic and post-earthquake phases.

A total of 1131 pediatric patients aged 0–18 years who were hospitalized during the study period and had at least one positive urine culture were included in the analysis. Only hospitalized children with a clinical diagnosis of active UTI were eligible for inclusion. Patients without compatible clinical findings or those considered to have asymptomatic bacteriuria were not hospitalized and therefore were not included in the study. UTI was defined by the presence of significant bacteriuria in urine cultures obtained according to standard microbiological procedures. Only culture-confirmed cases were evaluated. To ensure statistical independence and avoid duplication bias, a deduplication strategy was applied whereby only the first clinically significant positive urine culture and corresponding isolate per patient within the study period were included. Repeat cultures and subsequent isolates from the same patient were excluded from the analysis.

Demographic and clinical data, including age, sex, date of hospital admission, isolated uropathogens, and antibacterial susceptibility results, were extracted retrospectively from hospital laboratory and clinical information systems. Patients aged over 18 years at the time of admission, those with incomplete medical records, or urine cultures considered contaminated were excluded from the study.

The study was conducted in accordance with the ethical principles of the Declaration of Helsinki and was approved by the Malatya Turgut Özal University Non-Interventional Clinical Research Ethics Committee (Decision No: 56, Date: April 09, 2025). Due to the retrospective nature of the study, the requirement for informed consent was waived. Patient confidentiality was strictly maintained by anonymizing all data prior to analysis and using coded identifiers.

### Microbiological culture and isolate identification

Urine samples were collected using age-appropriate techniques in accordance with standard clinical practice. For children who were not toilet trained, urine specimens were obtained using sterile urine collection bags or catheterization when clinically indicated. In toilet-trained children, clean-catch midstream urine samples were collected. All specimens were transported promptly to the microbiology laboratory for processing.

Urine samples were inoculated onto 5% sheep blood agar and Eosin Methylene Blue (EMB) agar media (OR-BAK, Türkiye) using calibrated loops and incubated aerobically at 37 °C for 18–24 h. Following incubation, colony growth was assessed semiquantitatively. Significant bacteriuria was defined as ≥ 10^5^ colony-forming units (CFU)/mL for midstream urine samples and ≥ 10^3^ CFU/mL for catheter-obtained specimens. Samples not meeting these criteria or considered contaminated were excluded from the analysis.

Bacterial isolates were initially evaluated based on colony morphology, Gram staining characteristics, and conventional biochemical tests, including IMViC reactions when appropriate. Definitive identification at the species level was performed using the VITEK® 2 Compact automated identification system (bioMérieux, France), following the manufacturer’s instructions.

### Antibacterial susceptibility testing

Antibacterial susceptibility testing of all confirmed uropathogens was performed using the Kirby–Bauer disk diffusion method on Mueller–Hinton agar. The panel of antibacterial agents was selected based on those commonly used in pediatric clinical practice and included representatives of the major antibacterial classes. Antibacterial panels were determined according to organism-specific routine laboratory protocols and EUCAST recommendations. Susceptibility results were interpreted according to the European Committee on Antimicrobial Susceptibility Testing (EUCAST) clinical breakpoints. As EUCAST guidelines are updated annually (January releases), susceptibility interpretations for isolates obtained between 2020 and 2024 were performed using the EUCAST version current at the time of testing. For statistical analyses, isolates categorized as intermediate were classified as nonsusceptible. MDR was defined as nonsusceptibility to at least one agent in three or more distinct antibacterial categories, in accordance with internationally accepted criteria (Ahmed et al. 2025).

### Statistical analysis

All data were compiled and analyzed using the Statistical Package for the Social Sciences (SPSS) software, version 22.0 (IBM Corp., Chicago, IL, USA). Descriptive statistics were used to summarize the study population. Categorical variables were expressed as frequencies and percentages, while continuous variables were presented as mean ± standard deviation. Comparisons between groups were performed using the Pearson chi-square test for categorical variables. Statistical significance was defined as a two-tailed *p* value < 0.05.

## Results

### Baseline characteristics of the study population

**Fig. 1:**
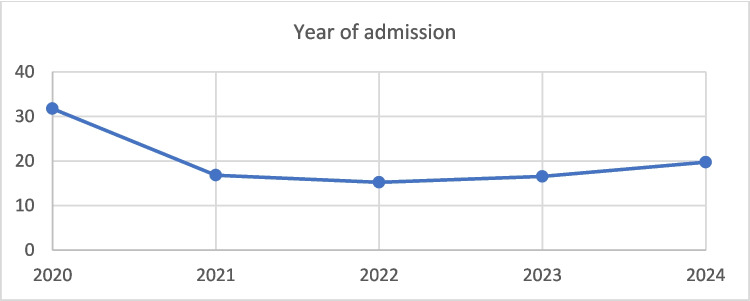
Yearly distribution of pediatric UTI hospitalizations (2020–2024)

The annual distribution of hospitalized pediatric UTI cases demonstrated a marked decline following 2020, reaching its lowest level in 2022, and subsequently showing a gradual increase through 2024 (Fig. 1). According to the predefined study periods, 63.75% of cases occurred during the COVID-19 period, 16.53% during the earthquake period, and 19.72% during the post-earthquake period.

The baseline demographic and clinical characteristics of the study population are summarized in Table 1. A total of 1131 hospitalized pediatric patients with UTIs were included. Overall, females slightly predominated, and most patients were between 12 and 60 months of age. The distribution of cases varied across participating provinces and study years. When stratified by predefined study periods, most cases were recorded during the COVID-19 pandemic period, followed by the post-earthquake and earthquake periods. Sex distribution differed significantly between study periods (*p* = 0.045), with a lower proportion of females observed in the post-earthquake period. In contrast, age group distribution did not significantly vary across periods (*p* = 0.538). Geographic distribution showed significant variation over time (*p* < 0.001).

**Table 1:** Baseline demographic and clinical characteristics of hospitalized children with UTI by study period

		Total	COVID-19	Earthquake	Post-earthquake	p^*^
		*n* (%)	*n* (%)	*n* (%)	*n* (%)	
**Sex**	Female	614 (54.29)	408 (56.59)	101 (54.01)	105 (47.09)	**0.045**
	Male	517 (45.71)	313 (43.41)	86 (45.99)	118 (52.91)	
**Age group**	< 12 months	344 (30.42)	223 (30.93)	53 (28.34)	68 (30.49)	0.538
	12–60 months	474 (41.91)	298 (41.33)	75 (40.11)	101 (45.29)	
	> 60 months	313 (27.67)	200 (27.74)	59 (31.55)	54 (24.22)	
**Province**	Adıyaman	294 (25.99)	173 (23.99)	56 (29.95)	65 (29.15)	** < 0.001**
	Malatya	364 (32.18)	262 (36.34)	62 (33.16)	40 (17.94)	
	Kahramanmaraş	473 (41.82)	286 (39.67)	69 (36.90)	118 (52.91)	

### Distribution of isolated microorganisms

Among the 1131 urine culture-positive isolates, 1013 (89.57%) were Gram-negative bacteria and 117 (10.43%) were Gram-positive bacteria. Gram-negative organisms constituted the majority of the isolates.

Within the Gram-negative group, members of the order Enterobacterales accounted for 84.72% of all isolates. *E. coli* was the most frequently isolated pathogen (59.86%), followed by *Klebsiella *spp. (18.92%), *Proteus *spp. (4.60%), *Enterobacter *spp. (0.80%), *Morganella morganii* (0.27%), *Citrobacter *spp. (0.18%), and *Serratia *spp. (0.09%). Nonfermentative Gram-negative bacteria constituted 4.86% of the isolates, with *Pseudomonas *spp. accounting for 4.33% and *Acinetobacter *spp. for 0.53%.

Among Gram-positive bacteria, *Enterococcus *spp. were the most frequently identified organisms, comprising 8.05% of the isolates. Coagulase-positive staphylococci accounted for 0.88%, with methicillin-resistant *S. aureus* (MRSA) and methicillin-susceptible *S. aureus* (MSSA) each representing 0.44%. Coagulase-negative staphylococci were detected in 0.97% of isolates, while beta-hemolytic streptococci accounted for 0.53%. The overall distribution of isolated microorganisms is summarized in Table 2.

**Table 2:** Distribution of microorganisms isolated from urine cultures in UTI pediatric population

		*n*	%
Gram-positive bacteria	**Coagulase-positive staphylococci**	**10**	**0.88**
	MRSA (methicillin-resistant *S. aureus*)	5	0.44
	MSSA (methicillin-susceptible *S. aureus*)	5	0.44
	**Coagulase-negative staphylococci**	**11**	**0.97**
	MSSE (methicillin-susceptible *S. epidermidis*)	3	0.27
	MRSE (methicillin-resistant *S. epidermidis*)	3	0.27
	MSS *S. haemolyticus*	3	0.27
	MRS *S. haemolyticus*	2	0.18
	***Enterococcus* spp.**	**91**	**8.05**
	**Beta-hemolytic streptococci**	**6**	**0.53**
Gram-negative bacteria	**Enterobacterales**	**958**	**84.72**
	*Enterobacter* spp.	9	0.80
	*Escherichia coli*	677	59.86
	*Klebsiella* spp.	214	18.92
	*Proteus* spp.	52	4.60
	*Serratia* spp.	1	0.09
	*Morganella morganii*	3	0.27
	*Citrobacter* spp.	2	0.18
	**Nonfermentative Gram-negative bacteria**	**55**	**4.86**
	*Acinetobacter* spp.	6	0.53
	*Pseudomonas* spp.	49	4.33

Table 3 presents the distribution of uropathogenic microorganisms isolated from pediatric urine samples according to sex and age groups. A statistically significant difference was observed in the distribution of microorganisms among age groups (*p* < 0.001), indicating a significant association between patient age and isolated uropathens.

**Table 3:** Distribution of uropathogenic microorganisms according to sex and age groups in pediatric patients

Organism	Sex		Age		
	Female	Male	< 12 months	12–60 months	> 60 months
Coagulase-positive staphylococci	5 (0.8)	5 (1.0)	3 (0.9)	6 (1.3)	1 (0.3)
Coagulase-negative staphylococci	5 (0.8)	6 (1.2)	2 (0.6)	7 (1.5)	2 (0.6)
*Enterococcus *spp.	54 (8.8)	37 (7.2)	40 (11.6)	30 (6.3)	21 (6.7)
*Streptococcus *spp.	1 (0.2)	5 (1.0)	1 (0.3)	4 (0.8)	1 (0.3)
*Enterobacter *spp.	4 (0.7)	5 (1.0)	5 (1.5)	3 (0.6)	1 (0.3)
*Escherichia coli*	394 (64.2)	283 (54.7)	189 (54.9)	264 (55.7)	224 (71.6)
*Klebsiella *spp.	109 (17.8)	105 (20.3)	65 (18.9)	114 (24.1)	35 (11.2)
*Proteus *spp.	19 (3.1)	33 (6.4)	19 (5.5)	22 (4.6)	11 (3.5)
*Serratia *spp.	1 (0.2)	NA	1 (0.3)	NA	NA
*Morganella morganii*	NA	3 (0.6)	2 (0.6)	NA	1 (0.3)
*Citrobacter *spp.	1 (0.2)	1 (0.2)	2 (0.6)	NA	NA
*Acinetobacter *spp.	2 (0.3)	4 (0.8)	3 (0.9)	3 (0.6)	NA
*Pseudomonas *spp.	19 (3.1)	30 (5.8)	12 (3.5)	21 (4.4)	16 (5.1)
p^*^	**0.007**		** < 0.001**		

### Distribution of uropathogens across study periods

**Fig. 2:**
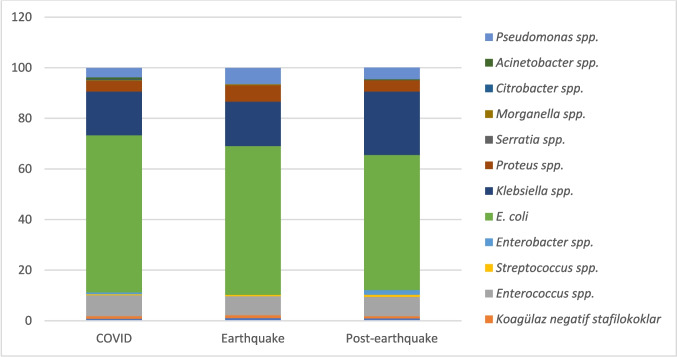
Distribution of uropathogenic microorganisms isolated from pediatric urine samples across the COVID-19, earthquake, and post-earthquake periods

The distribution of microorganisms isolated from urine samples of pediatric patients during the COVID-19 period, the earthquake period, and the post-earthquake period is presented in Table 4. *E. coli* was the predominant uropathogen across all periods, with isolation rates of 62.1% (*n* = 448) during the COVID-19 period, 58.8% (*n* = 110) during the earthquake period, and 53.4% (*n* = 119) in the post-earthquake period. *Klebsiella* spp*.* was the second most frequently isolated pathogen, showing a notable increase in the post-earthquake period (25.1%, *n* = 56) compared with the COVID-19 (17.3%, *n* = 125) and earthquake periods (17.6%, *n* = 33). *Enterococcus *spp. consistently ranked third, with comparable isolation rates across periods (7.5–8.3%). An increase in the isolation rates of *Proteus *spp. and *Pseudomonas *spp. was observed during the earthquake period (6.4% for each), followed by a decrease in the post-earthquake period (4.5%). Coagulase-positive and coagulase-negative staphylococci, streptococci, and other less common pathogens were detected at low frequencies throughout the study periods, while certain microorganisms, including *Enterobacter* spp., *Serratia* spp., *and Citrobacter *spp., were identified only in specific periods (Fig. 2).

**Table 4:** Distribution of uropathogenic microorganisms isolated from pediatric urine samples across the COVID-19, earthquake, and post-earthquake periods

Organism	COVID-19		Earthquake		Post-earthquake	
	*n*	%	*n*	%	*n*	%
Coagulase-positive staphylococci	6	0.8	2	1.1	2	0.9
Coagulase-negative staphylococci	7	1.0	2	1.1	2	0.9
*Enterococcus *spp.	60	8.3	14	7.5	17	7.6
*Streptococcus *spp.	3	0.4	1	0.5	2	0.9
*Enterobacter *spp.	5	0.7	NA	NA	4	1.8
*Escherichia coli*	448	62.1	110	58.8	119	53.4
*Klebsiella *spp.	125	17.3	33	17.6	56	25.1
*Proteus *spp.	30	4.2	12	6.4	10	4.5
*Serratia *spp.	1	0.1	NA	NA	NA	NA
*Morganella morganii*	2	0.3	1	0.5	NA	NA
*Citrobacter *spp.	2	0.3	NA	NA	NA	NA
*Acinetobacter *spp.	5	0.7	NA	NA	1	0.4
*Pseudomonas *spp.	27	3.7	12	6.4	10	4.5

^*^NA indicates that the microorganism was not isolated during the corresponding period

### AMR patterns of Gram-negative microorganisms

The AMR rates of Gram-negative microorganisms are presented in Table 5. Among *E. coli* isolates, resistance rates were 68.67% for ampicillin, 46.27% for ceftriaxone, and 45.31% for ceftazidime, while lower resistance rates were observed for amikacin (8.45%) and imipenem (7.30%).

**Table 5:** Antibacterial resistance rates of Gram-negative microorganisms, *n* (%)

	*E. coli*	*Klebsiella *spp.	*Pseudomonas *spp.	*Acinetobacter *spp.
Amikacin	56 (8.45)	53 (25.60)	2 (4.08)	2 (33.33)
Ampicillin	423 (68.67)	95 (95.00)	21 (91.30)	1 (100.00)
Gentamicin	104 (16.72)	59 (32.78)	6 (20.00)	4 (66.67)
Imipenem	46 (7.30)	41 (20.20)	23 (50.00)	2 (33.33)
Colistin	NA	NA	NA	NA
Cefoperazone–sulbactam	NA	1 (100.00)	NA	NA
Ceftazidime	227 (45.31)	76 (54.29)	17 (47.22)	2 (100.00)
Ceftazidime–avibactam	15 (19.74)	10 (34.48)	NA	NA
Ceftriaxone	248 (46.27)	105 (55.85)	18 (85.71)	1 (100.00)
Ciprofloxacin	124 (21.31)	51 (28.81)	9 (24.32)	6 (100.00)
Tigecycline	23 (10.09)	16 (19.75)	7 (53.85)	NA
Trimethoprim–sulfamethoxazole	238 (36.12)	86 (41.15)	26 (96.30)	3 (50.00)

*Klebsiella *spp. isolates showed resistance rates of 95.00% to ampicillin, 55.85% to ceftriaxone, and 54.29% to ceftazidime. Imipenem resistance among *Klebsiella *spp. was detected in 20.20% of isolates. No resistance to colistin was detected in either *E. coli* or *Klebsiella *spp. isolates (0.00%); however, colistin susceptibility testing was not performed for all isolates.

Among nonfermentative Gram-negative bacteria, *Acinetobacter *spp. demonstrated resistance rates of 100.00% to ampicillin, ceftazidime, ceftriaxone, and ciprofloxacin. Resistance to gentamicin and trimethoprim–sulfamethoxazole was observed in 66.67% and 50.00% of isolates, respectively.

*Pseudomonas spp*. isolates exhibited resistance rates of 91.30% to ampicillin, 85.71% to ceftriaxone, and 96.30% to trimethoprim–sulfamethoxazole. No resistance was detected to colistin, cefoperazone–sulbactam, or ceftazidime–avibactam in either non-fermentative Gram-negative bacterial group (0.00%).

Overall, resistance patterns across Gram-negative isolates revealed substantial variability between species and antibiotic classes. These distribution patterns and clustering tendencies are visually summarized in Table 6 (heat map).

**Table 6:**
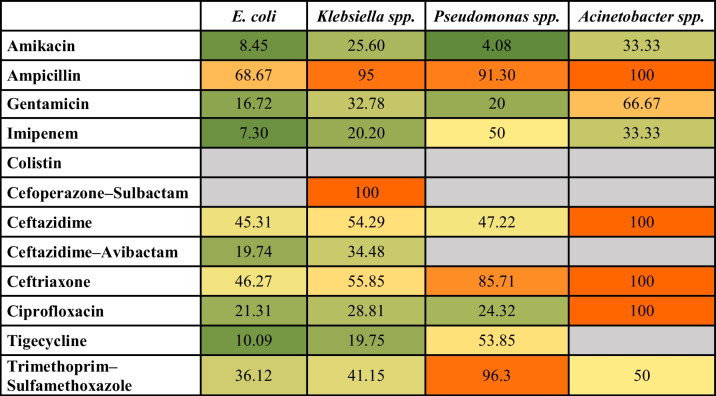
Heat map showing antibacterial resistance patterns among Gram-negative isolates

### Antibacterial resistance patterns of Gram-negative bacteria across study periods

Table 7 presents the comparison of antibacterial resistance rates among Gram-negative bacteria (*E. coli*, *Klebsiella *spp.,* Proteus *spp., and* Pseudomonas *spp.) during the COVID-19, earthquake, and post-earthquake periods. In *E. coli* isolates, ampicillin resistance increased from 63.26% during the COVID-19 period to 77.00% during the earthquake period and further to 81.90% in the post-earthquake period (*p* < 0.001). Ceftazidime resistance rates were 39.46%, 50.59%, and 63.10% across the respective periods, showing a statistically significant difference (*p* < 0.001). Ceftriaxone resistance was 41.99% during the COVID-19 period, increased to 61.29% during the earthquake period, and slightly decreased to 53.76% in the post-earthquake period (*p* = 0.005). Trimethoprim–sulfamethoxazole resistance increased from 32.27% during the COVID-19 period to 48.00% during the earthquake period and was 40.34% in the post-earthquake period (*p* = 0.007). No statistically significant differences were observed between periods for the remaining antibacterial agents (*p* > 0.05).

**Table 7:** Comparison of antibacterial resistance rates among gram-negative bacteria during the COVID-19, earthquake, and post-earthquake periods, *n* (%)

	*E. coli*				*Klebsiella spp.*				*Proteus spp.*				*Pseudomonas spp.*			
	COVID-19	Earthquake	Post-earthquake	p^*^	COVID-19	Earthquake	Post-earthquake	p*	COVID-19	Earthquake	Post-earthquake	p*	COVID-19	Earthquake	Post-earthquake	p^*^
Amikacin	37 (8.31)	4 (4.00)	15 (12.71)	0.069	31 (25.62)	10 (31.25)	12 (22.22)	0.651	3 (10.34)	0.00	2 (22.22)	0.268	1 (3.70)	1 (8.33)	0.00	0.702
Ampicillin	260 (63.26)	77 (77.00)	86 (81.90)	** < 0.001**	54 (94.74)	16 (94.12)	25 (96.15)	0.947	11 (40.74)	6 (54.55)	6 (66.67)	0.344	10 (90.91)	6 (100.00)	5 (83.33)	0.590
Gentamicin	66 (16.22)	11 (11.11)	27 (23.28)	0.053	31 (29.25)	10 (35.71)	18 (39.13)	0.460	4 (14.29)	6 (54.55)	4 (50.00)	**0.016**	2 (11.76)	2 (22.22)	2 (50.00)	0.218
Imipenem	27 (6.38)	9 (8.82)	10 (9.52)	0.440	22 (18.64)	12 (37.50)	7 (13.21)	**0.021**	12 (48.00)	7 (70.00)	7 (87.50)	0.120	12 (46.15)	6 (54.55)	5 (55.56)	0.853
Colistin	NT	NT	NT	-	NT	NT	NT	-	NT	NT	NT	-	NT	NT	NT	-
Cefoperazone–sulbactam	0.00	0.00	0.00	-	1 (100.00)	0	0.00	-	0.00	0.00	0.00	-	0.00	0.00	0.00	-
Ceftazidime	131 (39.46)	43 (50.59)	53 (63.10)	** < 0.001**	42 (53.16)	14 (58.33)	20 (54.05)	0.905	1 (3.85)	2 (18.18)	1 (12.50)	0.256	8 (40.00)	3 (30.00)	6 (100.00)	**0.014**
Ceftazidime/avibactam	5 (13.51)	2 (16.67)	8 (29.63)	0.267	6 (37.50)	3 (75.00)	1 (11.11)	0.077	0.00	0.00	0.00	-	0.00	0.00	0.00	-
Ceftriaxone	1690 (41.99)	38 (61.29)	50 (53.76)	**0.005**	60 (54.55)	19 (70.37)	26 (50.98)	0.238	2 (10.53)	1 (12.50)	4 (57.14)	**0.037**	9 (90.00)	5 (100.00)	4 (66.67)	0.402
Ciprofloxacin	79 (20.84)	19 (19.39)	26 (24.76)	0.603	31 (30.39)	8 (29.63)	12 (25.00)	0.789	4 (17.39)	1 (10.00)	0.00	0.658	4 (21.05)	1 (10.00)	4 (50.00)	0.184
Tigecycline	15 (11.54)	2 (7.69)	6 (8.33)	0.701	9 (18.37)	4 (36.36)	3 (14.29)	0.338	0.00	0.00	0.00	-	3 (50.00)	2 (66.67)	2 (50.00)	0.879
Trimethoprim–sulfamethoxazole	142 (32.27)	48 (48.00)	48 (40.34)	**0.007**	48 (39.34)	15 (45.45)	23 (42.59)	0.793	11 (37.93)	8 (66.67)	5 (50.00)	0.240	15 (93.75)	7 (100.00)	4 (100.00)	0.700

^*^Chi-square test was applied

When antibacterial resistance rates of *Klebsiella *spp. isolates were evaluated across the study periods, a statistically significant difference was observed only for imipenem. Imipenem resistance increased from 18.64% during the COVID-19 period to 37.50% during the earthquake period and subsequently decreased to 13.21% in the post-earthquake period (*p* = 0.021). No statistically significant differences were detected for the other antibacterial agents (*p* > 0.05).

In *Proteus *spp., gentamicin resistance increased markedly from 14.29% during the COVID-19 period to 54.55% during the earthquake period and remained high in the post-earthquake period (50.00%), with a statistically significant difference between periods (*p* = 0.016). Similarly, ceftriaxone resistance increased from 10.53% during the COVID-19 period to 12.50% during the earthquake period and showed a pronounced increase in the post-earthquake period (57.14%), resulting in a significant difference across periods (*p* = 0.037).

Among *Pseudomonas *spp. isolates, a statistically significant difference between periods was observed only for ceftazidime resistance. Ceftazidime resistance was 40.00% during the COVID-19 period, decreased to 30.00% during the earthquake period, and increased to 100.00% in the post-earthquake period (*p* = 0.014). No statistically significant differences were observed for the remaining antibacterial agents (*p* > 0.05).

NT: not tested.

For *Acinetobacter *spp. isolates, no statistically significant differences in antibacterial resistance rates were observed between the study periods for any antibacterial agent (*p* > 0.05). During the COVID-19 period, resistance rates to amikacin, gentamicin, and imipenem were 20%, 60%, and 20%, respectively. No *Acinetobacter *spp. isolates were detected during the earthquake period. In the post-earthquake period, resistance to ampicillin, ceftazidime, ceftriaxone, and ciprofloxacin was 100%, whereas no resistance was observed to colistin, cefoperazone–sulbactam, ceftazidime–avibactam, or tigecycline in any period.

For *Proteus *spp. isolates, ampicillin resistance increased across the COVID-19, earthquake, and post-earthquake periods (40.74%, 54.55%, and 66.67%, respectively), although this increase was not statistically significant (*p* = 0.344). Imipenem resistance also increased from 48.00% during the COVID-19 period to 70.00% during the earthquake period and 87.50% in the post-earthquake period; however, this difference did not reach statistical significance (*p* = 0.120). No statistically significant differences were observed for resistance to the remaining antibacterial agents (*p* > 0.05).

In *Serratia *spp., isolates identified during the COVID-19 period showed resistance rates of 100% to ampicillin, gentamicin, ceftazidime, ceftriaxone, and ciprofloxacin, while no resistance was detected to amikacin, imipenem, colistin, cefoperazone–sulbactam, ceftazidime–avibactam, tigecycline, or trimethoprim–sulfamethoxazole (0%). No *Serratia *spp. isolates were detected during the earthquake or post-earthquake periods; therefore, statistical comparisons between periods could not be performed.

For *Citrobacter *spp., isolates identified during the COVID-19 period exhibited resistance rates of 100% to amikacin, ampicillin, gentamicin, imipenem, ceftazidime, ceftriaxone, and trimethoprim–sulfamethoxazole, while ciprofloxacin resistance was 50%. No resistance was detected to colistin, cefoperazone–sulbactam, ceftazidime–avibactam, or tigecycline (0%). No *Citrobacter *spp. isolates were identified during the earthquake or post-earthquake periods, precluding interperiod statistical analysis.

In *Morganella *spp., isolates detected during the COVID-19 period showed 100% resistance to ampicillin, while resistance to imipenem and trimethoprim–sulfamethoxazole was 50%. During the earthquake period, resistance to both ampicillin and imipenem reached 100%. No *Morganella *spp. isolates were detected during the post-earthquake period. Differences in resistance rates between periods were not statistically significant (*p* = 0.386).

### Distribution MDR uropathogens isolated from pediatric urine samples

MDR uropathogens were identified among both Gram-positive and Gram-negative isolates (Table 8). Among Gram-negative bacteria, MDR rates were highest in *Serratia *spp. and *Citrobacter *spp., with all detected isolates exhibiting MDR profiles (100%). High MDR proportions were also observed in *Enterobacter *spp. (66.7%), *Morganella morganii* (66.7%), and *Pseudomonas *spp. (57.1%). *Klebsiella *spp. demonstrated a considerable MDR rate (38.3%), whereas *E. coli,* despite being the most frequently isolated pathogen, showed a comparatively lower MDR rate of 25.6%. MDR was also detected in *Proteus *spp. (17.3%) and *Acinetobacter *spp. (33.3%). Among Gram-positive bacteria, MDR was identified in *Enterococcus *spp. (13.2%) and beta-hemolytic streptococci (16.7%). Overall, MDR uropathogens were predominantly observed among Gram-negative organisms, particularly within the Enterobacterales group and nonfermentative Gram-negative bacteria.

**Table 8:** Distribution of MDR uropathogens isolated from pediatric urine samples

	Organism	MDR	
		*n*	%
Gram-positive bacteria	*Enterococcus *spp.	12	13.2
	Beta-hemolytic streptococci	1	16.7
Gram-negative bacteria	*Enterobacter *spp.	6	66.7
	*Escherichia coli*	173	25.6
	*Klebsiella *spp.	82	38.3
	*Proteus* spp*.*	9	17.3
	*Serratia *spp.	1	100.0
	*Morganella morganii*	2	66.7
	*Citrobacter *spp.	2	100.0
	*Acinetobacter *spp.	2	33.3
	*Pseudomonas* spp.	28	57.1

Univariate and multivariable analyses of factors associated with multidrug resistance are presented in Table 9. In univariate analyses, province, sex, age group, and study period were significantly associated with MDR (all *p* < 0.05), whereas gram staining was not. However, in multivariable logistic regression analysis adjusting for province, sex, age group, study period, and gram staining, study period was not independently associated with MDR. Compared with the COVID-19 period, neither the earthquake period (adjusted OR [aOR] 1.02, 95% CI 0.68–1.54; *p* = 0.910) nor the post-earthquake period (aOR 1.38, 95% CI 0.96–1.96; *p* = 0.076) showed a statistically significant association with MDR. In contrast, marked center-level differences were observed. Using Malatya as the reference center, the odds of MDR were substantially higher in Kahramanmaraş (aOR 16.26, 95% CI 8.36–31.60; *p* < 0.001) and Adıyaman (aOR 56.09, 95% CI 28.68–109.74; *p* < 0.001). Additionally, Gram-negative isolates were independently associated with higher odds of MDR compared to gram-positive isolates (aOR 2.87, 95% CI 1.77–4.66; *p* < 0.001).

**Table 9:** Univariate and multivariable analyses of factors associated with multidrug resistance

		Presence of multidrug resistance (MDR)		OR (95% CI)	p^**^
		*n* (%)	p^*^		
Province	Malatya	11 (3.02)	** < 0.001**	Ref	** < 0.001**
	Maraş	154 (32.56)		16.26 (8.36–31.60)	** < 0.001**
	Adıyaman	169 (57.48)		56.09 (28.68–109.74)	** < 0.001**
Sex	Female	165 (26.87)	**0.033**	Ref	0.417
	Male	169 (32.69)		0.88 (0.65–1.19)	
Age group	> 60 months	70 (22.36)	**0.005**	Ref	0.606
	12–60 months	152 (32.07)		0.91 (0.61–1.36)	0.659
	< 12 months	112 (32.56)		0.80 (0.52–1.24)	0.322
Study period	COVID	191 (26.49)	**0.001**	Ref	0.196
	Earthquake	55 (29.41)		1.02 (0.681–1.54)	0.910
	Post-earthquake	88 (39.46)		1.38 (0.96–1.96)	0.076
Gram stain	Gram-positive	29 (24.58)	0.212	Ref	** < 0.001**
	Gram-negative	305 (30.11)		2.87 (1.77–4.66)	

^*^Univariate (chi-square)

^**^Adjusted *p* (logistic regression)

## Discussion

This multicenter retrospective study evaluated temporal changes in uropathogen distribution and AMR patterns among hospitalized pediatric patients with UTIs across three provinces in eastern Türkiye during two major public health crises: the COVID-19 pandemic and the 2023 Kahramanmaraş earthquakes. By comparing the pandemic, earthquake, and post-earthquake periods, the study provides a real-world perspective on how large-scale disruptions to healthcare systems may influence pediatric UTI epidemiology and resistance dynamics.

Consistent with prior reports, Gram-negative bacteria accounted for the majority of uropathogens, with *E. coli* remaining the predominant causative agent throughout all study periods (Afsharipoor et al. 2025; Perween et al. 2022; Al Momani et al. 2025; Lin et al. 2025). Despite its continued dominance, a gradual decline in the proportion of *E. coli* isolates was observed from the pandemic to the post-earthquake period. In contrast, *Klebsiella *spp. demonstrated a noticeable increase, particularly following the earthquake, suggesting a potential shift toward more opportunistic and possibly healthcare-associated pathogens under conditions of healthcare system strain and altered clinical practices (Li et al. 2025).

The transient rise in *Proteus *spp. and *Pseudomonas *spp. observed during the earthquake period may reflect contextual changes in healthcare delivery and patient populations under emergency conditions. Factors such as emergency healthcare settings, overcrowding, compromised infection control measures, and increased exposure to broad-spectrum antibacterial agents may represent plausible mechanisms; however, these variables were not directly assessed in the present study. Notably, *Proteus *spp. and *Pseudomonas *spp. are commonly associated with complicated UTIs, prolonged hospitalization, and prior antibacterial exposure. Similar shifts in pathogen distribution have been reported in post-disaster and crisis-affected healthcare environments, supporting the plausibility of these observations without implying causality (Watson et al. 2007).

Beyond changes in microbial distribution, a key finding of this study is the marked escalation of AMR among major uropathogens, particularly *E. coli*, *Klebsiella *spp.,* Proteus *spp., and *Pseudomonas *spp. Resistance to commonly prescribed empirical agents—including ampicillin, third-generation cephalosporins, and trimethoprim–sulfamethoxazole—increased substantially across the study periods, with the most pronounced elevations occurring during and after the earthquake. These trends may reflect the combined effects of pandemic-related antibacterial overuse and disaster-associated disruptions to antibacterial stewardship and diagnostic capacity.

The substantial burden of MDR uropathogens observed in this cohort is of particular concern. MDR was predominantly detected among Gram-negative organisms, especially *Klebsiella *spp. and nonfermentative bacteria such as *Pseudomonas *spp., which are known to limit therapeutic options and complicate clinical management. Although resistance to carbapenems and colistin remained relatively low overall, the increase in imipenem resistance among *Klebsiella *spp. during the earthquake period may represent an early indicator of emerging resistance pressure, particularly in the absence of effective stewardship interventions.

Taken together, these findings suggest that while *E. coli* continues to be the leading cause of pediatric UTIs, large-scale public health emergencies may be associated with shifts in pathogen ecology and AMR profiles. Such changes underscore the importance of context-specific empirical treatment strategies, supported by up-to-date local surveillance data, particularly during periods of healthcare system overload and in disaster-prone regions (Kouadio et al. 2012).

The resistance patterns observed in this study are biologically and clinically plausible within the context of the extraordinary circumstances following the 2023 Kahramanmaraş earthquakes. The twin earthquakes (magnitudes 7.7 and 7.6) were associated with widespread infrastructure damage and major disruptions to healthcare delivery across the region, affecting nearly 14 million individuals (Ozturk et al. 2024). Combined with pandemic-related changes in healthcare utilization, increased antibacterial consumption, compromised sanitation, overcrowded temporary shelters, and delays in medical care, these conditions may represent plausible contextual drivers influencing UTI epidemiology and AMR patterns among pediatric patients. However, these variables were not directly measured in the present study and should therefore be interpreted with caution.

UTIs are among the most common bacterial infections in neonates and young children and often present with nonspecific clinical manifestations, which may delay diagnosis and initiation of appropriate therapy. Prompt recognition and timely antibacterial treatment are therefore crucial to prevent serious complications, including bacteremia, meningitis, renal scarring, and long-term impairment of renal function. UTIs are reported more frequently in male infants—particularly uncircumcised boys and those with vesicoureteral reflux—whereas febrile UTIs constitute a substantial proportion of pediatric hospitalizations and are commonly associated with congenital anomalies of the kidney and urinary tract. Although acute kidney injury has been increasingly recognized as a complication of febrile UTIs in adult populations, its burden in children remains underreported and likely underestimated (Marzuillo et al. 2024; Heno et al. 2025).

Infectious complications following earthquakes have been extensively documented in pediatric populations. Yılmaz et al. reported wound infections in 58.3% and UTIs in 25% of pediatric earthquake victims, highlighting the vulnerability of children to infections in disaster settings (Yilmaz et al. 2024). Similarly, Doğantekin et al. demonstrated a high prevalence of pathogenic microorganisms among children living in tent cities, frequently accompanied by coinfections (Dogantekin et al. 2024), while Aykaç et al. reported polymicrobial infections in more than half of pediatric earthquake victims, with a predominance of Gram-negative bacilli (Aykac et al. 2024) Our findings are consistent with these reports and extend the existing literature by suggesting that disaster-related conditions not only increase susceptibility to Gram-negative infections but may also facilitate the emergence and dissemination of antibacterial-resistant uropathogens among hospitalized children.

During the COVID-19 pandemic, several studies reported a transient decline in pediatric UTI diagnoses, which has been associated with reduced healthcare utilization, delayed hospital admissions, and limited access to diagnostic services (Kuitunen et al. 2022; Liang et al. 2024). In line with these observations, our study identified notable temporal shifts in uropathogen distribution across the pandemic, earthquake, and post-earthquake periods. Although *E. coli* remained the predominant uropathogen throughout the study period, its relative frequency declined over time, whereas *Klebsiella *spp. showed a significant increase in the post-earthquake period. These temporal changes may reflect broader contextual alterations in healthcare delivery during large-scale disruptions, including changes in antibacterial prescribing practices, hospitalization patterns, and living conditions. However, these factors were not directly assessed and should therefore be interpreted with caution.

Sex- and age-specific differences in uropathogen distribution were also evident in our cohort. *E. coli* was the most frequently isolated pathogen in both sexes, while *Klebsiella *spp. were more frequently detected in boys, than in girls, potentially reflecting a higher prevalence of underlying urinary tract anomalies or factors related to circumcision status (Singh-Grewal et al. 2005). Children aged 12–60 months represented the largest subgroup of UTI cases and exhibited higher isolation rates of *Klebsiella* spp., *Staphylococcus *spp.—including both coagulase-positive and coagulase-negative species—and *Streptococcus *spp., which may be attributed to increased healthcare exposure and vulnerability during this developmental period. In contrast, *Proteus *spp*.* were more frequently isolated in male infants younger than 12 months, a finding that may be explained by the organism’s urease activity, which promotes urinary alkalinization and facilitates stone formation (Demirgan and Canpolat 2021). Collectively, these findings highlight the importance of incorporating age- and sex-specific considerations into the empirical management and follow-up of pediatric UTIs.

In line with these demographic and microbiological patterns, the overall etiological profile observed in the present multicenter study showed substantial concordance with the large-scale single-center pediatric UTI study conducted in Diyarbakır, a region characterized by widespread antibacterial use (Samanci and Pınarbaşi 2023). In both studies, Gram-negative bacteria predominated, with members of the order Enterobacterales accounting for the majority of isolates. Although *E. coli* remained the leading uropathogen in both cohorts, its prevalence was lower in our multicenter study (59.86%) compared with the Diyarbakır cohort (68.1%). In contrast, *Klebsiella *spp. were isolated more frequently in our population (18.92% vs. 12.6%), a difference that may reflect intercenter differences in patient referral patterns, prior antibacterial exposure, hospitalization rates, and healthcare utilization during periods of systemic strain. Consistent with previous reports, *E. coli* infections were more common among female patients, whereas non-*E. coli* uropathogens predominated in males, likely reflecting anatomical and functional differences in the pediatric urinary tract. Although nonfermentative Gram-negative bacteria accounted for a relatively small proportion of isolates in both datasets, the persistent detection of *Pseudomonas *spp. and *Acinetobacter *spp. in our multicenter cohort may indicate a higher burden of complicated infections or increased hospital-associated exposure. Collectively, these findings suggest that while the etiological landscape of pediatric UTIs remains broadly similar in regions with high antibacterial consumption, local and center-specific variations warrant careful consideration when developing empiric antibacterial treatment strategies informed by regional surveillance data.

Comparable trends have also been reported in regional studies from neighboring countries, supporting the external consistency of our findings. In a large pediatric cohort study from Iran, Nateghian et al. analyzed 1048 culture-positive urine samples and identified *E. coli* as the predominant uropathogen (77.6%), followed by *Klebsiella pneumoniae* (10.4%), *Pseudomonas aeruginosa* (2.4%), and *Enterococcus *spp. (2.4%) (Nateghian et al. 2021). Notably, the authors documented a progressive increase in *E. coli* resistance to multiple commonly used agents—including amikacin, ceftriaxone, ceftazidime, ciprofloxacin, trimethoprim–sulfamethoxazole, and imipenem—over the 2005–2010 period. Similar challenges were highlighted in a prospective pediatric study from India, where Ranjan et al. reported marked heterogeneity in antibacterial susceptibility patterns, complicating standardized empirical therapy. In that cohort, *E. coli* and *Klebsiella *spp. retained the highest susceptibility to carbapenems, particularly imipenem, meropenem, and ertapenem, while resistance to noncarbapenem agents remained substantial (Abhishek et al. 2025). In agreement with these regional observations, our study demonstrated a statistically significant increase in *E. coli* resistance to ampicillin, ceftriaxone, ceftazidime, and trimethoprim–sulfamethoxazole between 2020 and 2024. Furthermore, with the exception of tigecycline, resistance rates to nearly all tested antibacterial agents showed an upward trend—most prominently in the post-earthquake period—suggesting that large-scale healthcare disruptions may intensify preexisting AMR trajectories in pediatric uropathogens.

A Central European surveillance study conducted between 2011 and 2019 reported persistently high resistance to ampicillin and a gradual increase in cephalosporin resistance among *Klebsiella *spp., while susceptibility to aminoglycosides and fluoroquinolones largely remained preserved (Hrbacek et al. 2020). In contrast, our findings demonstrate a substantially more concerning resistance profile, particularly during the post-earthquake period. Ampicillin resistance reached critically high levels in *E. coli* (81.9%), *Klebsiella *spp. (96.15%), and *Proteus *spp. (66.67%). Moreover, ciprofloxacin resistance was notably elevated in *E. coli* (24.76%) and *Klebsiella *spp. (25%), accompanied by increased resistance to amikacin (*E. coli*: 12.71% and *Klebsiella *spp.: 22.22%), contrasting sharply with the high susceptibility rates (> 95%) reported in many European cohorts. Of particular concern, imipenem resistance exceeded 10% among major uropathogens, signaling a narrowing therapeutic window for severe pediatric UTIs. These marked disparities likely reflect differences in antibacterial stewardship policies, antibacterial accessibility, and the compounded effects of healthcare system disruption following large-scale natural disasters, underscoring the heightened vulnerability of low- and middle-resource settings to accelerated AMR.

Our findings also align with and extend our previous regional observations. In our earlier study conducted between 2016 and 2019 in Eastern Türkiye, predominantly involving adult patients, *E. coli* isolates retained high susceptibility to carbapenems and aminoglycosides, although resistance to ampicillin was already substantial (Erdoğan and Akbulut 2021). In contrast, the present pediatric cohort spanning 2020–2024 demonstrates a clear escalation in AMR, particularly to β-lactam antibacterial agents. Resistance rates to ampicillin (68.67%), ceftriaxone (46.27%), and ceftazidime (45.31%) were markedly higher than those previously reported, indicating a progressive erosion of the efficacy of commonly used agents over time. Although amikacin and imipenem continue to exhibit relatively low resistance rates, the upward trend relative to earlier data is concerning and highlights the growing AMR burden among pediatric uropathogens, likely exacerbated by pandemic- and disaster-related disruptions in healthcare delivery and antibacterial stewardship.

In the present multicenter study, MDR uropathogens were predominantly identified among Gram-negative bacteria, particularly within the Enterobacterales order and nonfermentative Gram-negative organisms. Strikingly high MDR rates were observed in *Serratia *spp. and *Citrobacter *spp., with all detected isolates exhibiting MDR phenotypes, while substantial MDR proportions were also noted in *Enterobacter *spp.,* Morganella morganii*, and *Pseudomonas *spp., underscoring their growing clinical relevance in pediatric UTIs. Although *E. coli* remained the most frequently isolated pathogen, its MDR rate (25.6%) was comparatively lower than those reported from regions with intense antibacterial pressure. For instance, studies from India have documented markedly higher MDR rates in *E. coli* (80.85%), *Enterococcus *spp. (84.6%), and *Klebsiella pneumoniae* (62.5%), with additional MDR detection in *P. aeruginosa* and *Proteus mirabilis* (Ahmed et al. 2025). The relatively lower MDR prevalence observed among *E. coli* and Gram-positive isolates in our cohort may reflect regional differences in antibacterial exposure and stewardship practices. Nevertheless, the considerable MDR rate observed in *Klebsiella *spp. (38.3%) supports its emerging role as a particularly challenging uropathogen in pediatric populations and highlights substantial geographic variability in MDR epidemiology, reinforcing the need for region-specific surveillance strategies to guide empiric therapy. Notably, multivariable analyses did not identify study period as an independent predictor of MDR, suggesting that observed variations may be influenced by center-level and microbiological determinants rather than temporal effects alone.

The multivariable analysis provides additional context for interpreting the observed MDR patterns. Although univariate comparisons suggested temporal differences, the study period was not independently associated with MDR after adjustment, indicating that apparent temporal variations may be partially explained by differences in pathogen distribution and center-specific characteristics. In contrast, substantial variability was observed between study centers, with higher odds of MDR in certain provinces. These differences could reflect heterogeneity in patient populations, healthcare utilization, local prescribing practices, or stewardship-related factors, particularly in the setting of healthcare system disruptions. Additionally, the independent association between Gram-negative organisms and MDR is consistent with well-established resistance dynamics reported for Enterobacterales and nonfermentative Gram-negative bacteria. Collectively, these findings suggest that MDR epidemiology in this context is likely influenced by multiple interacting determinants and should therefore be interpreted with caution.

The global inappropriate use and overprescription of antibacterial agents during the COVID-19 pandemic has substantially accelerated the emergence of AMR, complicating infection management in the post-pandemic era (Khoo et al. 2022). In disaster-affected regions, this challenge was further amplified by damage to healthcare infrastructure, disruptions in routine infection control practices, and the interruption of antibacterial stewardship programs. Under such conditions, empirical antibacterial use often becomes broader and less targeted, creating a favorable environment for the selection and dissemination of MDR organisms, particularly in vulnerable pediatric populations requiring hospitalization.

In Türkiye, approximately 12.5% of secondary- and tertiary-level healthcare facilities are located within the 11 provinces severely impacted by the February 6, 2023 Kahramanmaraş earthquakes. The disaster was associated with extensive structural damage, affecting 42 hospital buildings and creating substantial challenges for healthcare continuity and emergency response capacity. Although 77 field hospitals were rapidly established to mitigate infrastructure losses, healthcare delivery remained under intense strain due to overwhelming patient volumes, workforce shortages, and the personal and professional losses experienced by healthcare workers—especially in Malatya, Adıyaman, Kahramanmaraş, and Hatay (T.C.Cumhurbaşkanlığı, 2023; Çakın et al. 2024). These extraordinary circumstances may have contributed to delayed diagnoses, prolonged hospitalizations, increased exposure to broad-spectrum antibacterial agents, and altered infection control dynamics, collectively facilitating conditions favorable to the emergence of resistant pathogens.

Population displacement following the earthquakes further compounded these challenges. According to Turkish Statistical Institute data, nearly 3.45 million people migrated between provinces in 2023, with approximately 14.5% relocating due to natural disasters and emergencies. Residents from the earthquake-affected provinces migrated primarily for healthcare access, housing, education, and economic stability, thereby increasing healthcare demand in receiving regions while disrupting continuity of care in the affected areas (Türkiye Cumhuriyeti 2023; Düzenli 2024). Such large-scale population movements may have facilitated the dissemination of resistant microorganisms across regions and potentially altered local AMR profiles. Taken together, these findings emphasize that AMR in pediatric UTIs is not merely a microbiological phenomenon but a complex public health challenge shaped by pandemics, natural disasters, healthcare system resilience, and population mobility. Addressing this growing burden requires sustained regional surveillance, pediatric-focused antibacterial stewardship programs, and disaster-resilient healthcare strategies to preserve therapeutic options and protect vulnerable children from long-term infectious and renal complications.

## Strengths and Limitations

This study has several notable strengths. To our knowledge, it represents one of the most comprehensive multicenter investigations conducted in Türkiye examining pediatric UTIs and AMR patterns in the post-earthquake context. The participating centers were deliberately selected from provinces most severely affected by the February 6, 2023 earthquakes, providing a unique opportunity to evaluate AMR and UTI dynamics under consecutive large-scale public health disruptions, including the COVID-19 pandemic and earthquake-related healthcare disturbances. This intentional design enabled a focused assessment of regions exposed to overlapping systemic stressors.

Several limitations should also be considered. First, despite its severe earthquake impact, Hatay could not be included due to extensive healthcare infrastructure damage and the disruption of routine clinical services. Second, ESBL-producing organisms could not be consistently evaluated, as routine ESBL testing was not uniformly performed across centers. Third, the absence of pre-2020 baseline data restricts the interpretation of long-term secular resistance trends. Fourth, the retrospective design may have been associated with incomplete data capture and potential unmeasured confounding.

Additionally, although study center was incorporated into multivariable analyses, formal multilevel modeling was not performed; therefore, residual center-level clustering effects cannot be entirely excluded. Missing data were handled using available-case analyses without multiple imputation. Given the exploratory nature of the study, adjustments for multiple comparisons were not applied, and formal sensitivity analyses were not conducted. Furthermore, the classification of the entire year of 2023 as the “earthquake period,” while analytically pragmatic, may obscure finer temporal variations.

Finally, the study population comprised only hospitalized children, which may limit generalizability. Moreover, urine bag collection methods—particularly in younger patients—carry an inherent risk of contamination despite standard antiseptic precautions. These factors should be considered when interpreting resistance estimates. Notwithstanding these limitations, the multicenter design, large sample size, and distinctive temporal framework provide valuable descriptive insights into AMR patterns during major public health disruptions.

## Conclusion

This multicenter study provides a comprehensive overview of the microbial spectrum and AMR patterns of pediatric UTIs in eastern Türkiye across the COVID-19 pandemic, the 2023 Kahramanmaraş earthquakes, and the post-earthquake period. *E. coli* remained the predominant uropathogen throughout all study periods, followed by *Klebsiella *spp. and *Enterococcus *spp., while notable age-, sex-, and period-specific variations were observed in the distribution of non-*E. coli* pathogens. Our findings suggest temporal increases in resistance to commonly used antibacterial agents, particularly among Gram-negative organisms, with comparatively higher resistance frequencies observed in the post-earthquake period for β-lactams, aminoglycosides, and trimethoprim–sulfamethoxazole among key uropathogens, including *E. coli, Klebsiella *spp.,* Proteus *spp., and *Pseudomonas *spp*.*

A substantial burden of multidrug-resistant uropathogens, predominantly among Enterobacterales and nonfermentative Gram-negative bacteria, was observed, posing persistent challenges for empiric treatment strategies in hospitalized children. Although carbapenems, colistin, and selected reserve agents generally preserved in vitro activity, the increasing reliance on these agents warrants caution given the risk of further resistance selection. Collectively, these findings underscore that AMR in pediatric UTIs constitutes not only a microbiological concern but also an important child health issue that may reflect the impact of healthcare system pressures during major public health disruptions. Continuous, region-specific surveillance, rational antibacterial prescribing, and strengthened pediatric antibacterial stewardship programs remain essential to preserve treatment efficacy, support evidence-based empiric therapy, and mitigate long-term morbidity in this vulnerable population.

## Data Availability

All source data for this work (or generated in this study) are available upon reasonable request.

## Declarations

### Declaration of AI and AI-assisted technologies in the writing process

During the preparation of this manuscript, the authors utilized the AI software ChatGPT to improve language clarity and enhance readability. Following this assistance, all content was thoroughly reviewed, edited, and verified by the authors, who take full responsibility for the accuracy, integrity, and final version of the publication.
